# Clinical predictors of an abnormal ultrasound in patients presenting with suspected nephrolithiasis

**DOI:** 10.12669/pjms.333.12651

**Published:** 2017

**Authors:** Salman Tahir Shafi, Roshina Anjum, Tahir Shafi

**Affiliations:** 1 Dr. Salman Tahir Shafi, FACP, FASN. Diplomat American Board of Internal Medicine and Nephrology, Department of Nephrology, Sharif Medical and Dental College, Sharif Medical City Road Jati Umra, Lahore, Pakistan; 2 Dr. Roshina Anjum, MBBS, Post Graduate Resident. Department of Nephrology, Sharif Medical and Dental College, Sharif Medical City Road Jati Umra, Lahore, Pakistan; 3 Prof. Dr. Tahir Shafi, FCPS. Diplomat American Board of Internal Medicine and Nephrology, Department of Nephrology, Sharif Medical and Dental College, Sharif Medical City Road Jati Umra, Lahore, Pakistan

**Keywords:** Nephrolithiasis, Predictors, Ultrasound

## Abstract

**Objective:**

To determine any clinical features associated with an abnormal ultrasound in patients with suspected nephrolithiasis in an out-patient setting.

**Methods:**

The study design was cross-sectional in nature. The study was conducted at an out-patient nephrology department of a tertiary care facility over a 3 month period. Patients included in the study were 18-80 years old, who presented with unilateral flank or costovertebral angle pain with or without other clinical features suggestive of renal or ureteric calculus based on clinician’s judgement. Every patient’s history was reviewed to obtain information on age, gender, location and radiation of pain, onset, severity and nature of pain, associated urinary and systemic symptoms and past history of nephrolithiasis. An ultrasound was considered to be abnormal if there was documented presence of renal or ureteric stone and/or unilateral hydronephrosis.

**Results:**

A total of 209 patients were included in the study. Of these patients, 126 (60.3%) were males and 83 (39.7%) were females, 60 (28.7%) had prior history of nephrolithiasis. Ultrasound was abnormal in 110 patients (52.9%). On a multivariate logistic regression analysis, only past history of nephrolithiasis (OR 3.3, 95% CI 1.65-6.7) was associated with an abnormal ultrasound.

**Conclusion:**

In the absence of any significant clinical predictors use of ultrasound is justified in patients with suspected nephrolithiasis especially in those with prior history of stones.

## INTRODUCTION

Pain from nephrolithiasis is a common reason for visiting health care facility.^1^ CT scan is more sensitive in detection of nephrolithiasis.^2^,^3^ However, CT scan is associated with radiation exposure and a large randomized clinical trial has shown that using ultrasound initially results in no significant difference in high risk diagnoses, adverse events, hospitalizations, emergency department visits and pain scores. There was lower cumulative radiation exposure with ultrasound.^3^ Ultrasound can be used as an initial test in a patient with suspected nephrolithiasis avoiding expense and radiation exposure of CT scan.^3^-^5^

It has been observed in clinical practice, that patients presenting with flank pain in an out-patient setting are worried about possibility of renal or ureteric stone and often demand renal ultrasound to exclude that possibility. Even though ultrasound is cheaper than CT scan, it still adds cost and increases work burden of radiology department. It is unclear whether there are any reliable clinical features which can predict abnormal ultrasound findings in patients presenting in an out-patient setting with flank pain and suspicion of nephrolithiasis, thus obviating need for ultrasound in some patients.

In a study by Moore et al., STONE score based on five factors (male sex, short duration of pain, non-black race, nausea or vomiting and microscopic hematuria) was predictive of uncomplicated ureteric stone on non-contrast CT scan in patients presenting in an emergency setting.^6^ However, there is limited information on clinical predictors of an abnormal ultrasound in patients presenting with flank pain and suspected nephrolithiasis in an out-patient rather than emergency setting.

The objective of this study was to determine the clinical predictors of an abnormal ultrasound in patients suspected to have nephrolithiasis who presented in an out-patient department.

## METHODS

The study was conducted at an out-patient nephrology department of a tertiary care facility. The study design was cross-sectional in nature. The study was conducted over a three month period from December 2016 till February 2017. Sampling methodology was non-probability consecutive sampling. The study was approved by institutional review board. Informed consent was obtained from each participating patient. Patients included in the study were 18-80 years old, who presented with unilateral flank or costovertebral angle pain with or without other clinical features suggestive of renal or ureteric calculus based on clinician’s judgement. Patients were excluded from the study if they were unwilling to undergo renal ultrasound. Every patient’s history was reviewed to obtain information on age, gender, location and radiation of pain, onset, severity and nature of pain, associated urinary and systemic symptoms. Patients were inquired about past history of nephrolithiasis, which was defined as history of renal or ureteric stone documented on radiological imaging or history of passage of stone in urine. Each patient was examined to document presence of costovertebral angle tenderness. Costovertebral angle tenderness was elicited by applying modest pressure with thumb on an area between 12^th^ rib and vertebral column on the same side where patient was complaining of pain.

All patients underwent renal ultrasound using Logiq P5 ultrasound machine (General Electric, Boston MA, USA) with a 3.5 MHz transducer. An ultrasound was considered abnormal if there was documented presence of renal or ureteric stone and/or unilateral hydronephrosis.

### Statistical Analysis

Continuous parametric variables were reported as means ± standard deviation and categorical variables were expressed as percentages. Categorical variables were compared using the chi-square test, and continuous variables were compared using t-test. Multivariate logistic regression analysis was done to determine predictors of an abnormal renal ultrasound. For multivariate analysis, all clinically relevant variables were included, and forward selection and likelihood ratios were used to determine the most efficient model. Adjusted odds ratios for all variables were calculated from the logistic regression analysis. All statistical analyses were performed using SPSS 20.0 (Chicago, IL USA). For all tests, p values of <0.05 were considered statistically significant.

## RESULTS

A total of 209 patients were included in the study. Of these patients, 126 (60.3%) were males and 83 (39.7%) were females, 60 (28.7%) had prior history of nephrolithiasis. Ultrasound was abnormal in 110 patients (52.9%). Demographic and clinical characteristics of all patients are shown in Table-I.

**Table-I:** Clinical characteristics of all patients.

*Clinical Characteristics*	*Mean (±SD) or Frequency (%)*
Mean Age in years	34.1±13.01
***Sex***	
Males	126 (60.3%)
Females	83 (39.7%)
***Location of pain***	
Flank Pain	156 (75.4%)
Costovertebral angle pain	53 (24.6%)
***Radiation***	
None	85 (48.8%)
Front	64 (30.6%)
Groin	50 (23.9%)
Genitalia	10 (4.7%)
***Onset of pain***	
Sudden	100 (47.8%)
Gradual	109 (52.2%)
***Severity of pain***	
Mild to Moderate	133 (63.6%)
Severe	76 (36.4%)
***Pain Character***	
Colicky	122 (58.4%)
Continuous	87 (41.6%)
***Urinary symptoms***	
None	56 (26.8%)
Dysuria	99 (47.4%)
Frequency/urgency	39 (18.7%)
Hematuria	15 (7.2%)
***Systemic symptoms***	
None	90 (43.1%)
Nausea, vomiting	109 (52.2%)
Fever	10 (4.7%)
***Aggravating factors***	
None	111 (53.1%)
Movement	67 (32.1%)
Rest	31 (14.8%)
***Past history of renal stones***	
Yes	60 (28.8%)
No	149 (71.2%)
***Findings on examination***	
None	152 (72.7%)
Costovertebral angle tenderness	57 (27.3%)

A comparison of clinical and demographic characteristics of patients with and without abnormal ultrasound findings is shown in Table-II. A multivariate logistic regression analysis of predictors of abnormal ultrasound was performed. Prior history of nephrolithiasis was the only variable significantly associated with an abnormal ultrasound (Adjusted odds ratio 3.3, 95% CI 1.67-6.5).

**Table-II:** A comparison of clinical characteristics of patients with and without abnormal ultrasound.

*Clinical characteristics*	*Normal Ultrasound N=99*	*Abnormal Ultrasound N=110*	*P value*
Mean Age in years	34.3±12.9	34.5±13.2	0.97
Male Sex	60 (47.2%)	66 (52.8%)	0.98
Flank pain	71 (45.5%)	85 (54.5%)	0.36
Radiation to Front/groin/genitalia	59 (42%)	64 (58%)	0.69
Sudden onset of pain	48 (48%)	52 (52%)	0.81
Severe pain	39 (51.3%)	37 (48.7%)	0.36
Colicky pain	60 (49.2%)	62 (50.8%)	0.48
Any urinary symptoms	74 (48.4%)	79 (51.6%)	0.62
Nausea, vomiting or fever	55 (46.2%)	64 (53.8%)	0.76
Aggravation by movement	29 (43.3%)	38 (56.7%)	0.74
Past history of nephrolithiasis	17 (28.3%)	43 (71.7%)	0.001
Costovertebral angle tenderness	26 (45.6%)	31 (54.4%)	0.79

## DISCUSSION

In our study, we found that ultrasound was abnormal in over half of patients who presented with flank pain and suspicion of nephrolithiasis. Only prior history of nephrolithiasis was significantly associated with an abnormal ultrasound.

Other studies have found similar or lower frequency of nephrolithiasis on imaging in patients with suggestive symptoms. Ureteric stones were found in 47.7% of all CT scans in one study,^7^ whereas confirmation of stone within six months was made in 1/3^rd^ of patients who underwent either ultrasound or CT scan as an initial imaging study.^3^

In our study, we used ultrasound as a reference investigation despite its lower sensitivity compared to CT scan. For example, sensitivity and specificity of ultrasound are 54%-57.3% and 73%-97.5% respectively in identifying nephrolithiasis^3^,^8^ and ultrasound has been found to be less accurate in detection of renal stones compared to CT scan.^9^ However, unilateral hydronephrosis in addition to finding of stone on ultrasound increases its sensitivity to 81.3-82.4% in identifying nephrolithiasis.^10^,^11^ We included finding of unilateral hydronephrosis with or without nephrolithiasis as a criteria for an “abnormal ultrasound”. Use of ultrasound as an initial imaging study is also justified based on several studies which have shown no difference in patient management and outcomes between ultrasound and CT scan in a patient with suspected nephrolithiasis.^3^,^12^-^15^ Ultrasound was found to be 97% sensitive in predicting need for surgical intervention when it showed a stone and/or hydronephrosis in patients presenting with renal colic.^12^ In other studies, rate of urological intervention was significantly lower^13^,^15^ or no patients required admission within 30 days^14^ in those with normal results on ultrasound.

Only prior history of nephrolithiasis was predictive of abnormal ultrasound finding in our study. In a study by Moore et al., STONE score based on five factors (male sex, short duration of pain, non-black race, nausea or vomiting and microscopic hematuria) was predictive of uncomplicated ureteric stone on non-contrast CT scan in patients presenting in an emergency setting.^6^ STONE score has been found to be valid in younger population as well.^16^ In our study, we didn’t identify any association between variables listed in STONE score and abnormal finding on ultrasound. There are several explanations for this. First, STONE score was derived based on findings on CT scan in patients visiting emergency department. Our study was based on an out-patient population, who underwent ultrasound rather than CT scan. In addition, we also included patients with renal stones rather than patients with ureteric calculi alone and we assessed hematuria based on patient’s history only rather than microscopic examination. Our study demographics were also different compared to STONE risk derivation study. Another observational multi-institutional external validation study has put a question mark on utility of STONE score as its sensitivity was found to be only 53% and specificity was 87% for ureteric stone in high risk group patients.^17^

### Limitations of the study

It was a single center study with sizeable but still limited study population. Though use of ultrasound is justified as an initial investigation based on existing literature, we didn’t have a CT scan for comparison, which is considered a gold standard for diagnosis of nephrolithiasis. In addition, there was no follow up data on further investigations, pain scores, emergency department visits or urological interventions in these patients.

## CONCLUSION

Ultrasound was found to be abnormal in over half of patients with suspected nephrolithiasis. In the absence of any reliable clinical predictors of abnormal findings on ultrasound with the exception of prior history of nephrolithiasis, we recommend that use of ultrasound is justified during initial evaluation of these patients. Patients with prior history of nephrolithiasis are more likely to have an abnormal ultrasound specifically vindicating use of ultrasound in these patients.

### Authors’ Contribution

**STS:** Data analysis, manuscript writing.

**RA:** Data collection, Data interpretation.

**TS:** Study design, manuscript review.
